# The Influence of Articulatory Suppression on Reading Among Chinese Children With Developmental Dyslexia: An Eye-Movement Study

**DOI:** 10.3389/fped.2021.758615

**Published:** 2021-11-25

**Authors:** Xiuhong Li, Weidong Li, Buyun Liu, Jinxin Zhang, Jingwen Ma, Chuanbo Xie, Jing Wu, Jin Jing

**Affiliations:** ^1^Department of Maternal and Child Health, School of Public Health, Sun Yat-sen University, Guangzhou, China; ^2^Department of Maternal and Child Health Information, Guangzhou Women and Children's Medical Center, Guangzhou, China; ^3^Division of Life Sciences and Medicine, University of Science and Technology of China, Hefei, China; ^4^Department of Medical Statistics and Epidemiology, School of Public Health, Sun Yat-sen University, Guangzhou, China; ^5^Guangdong Provincial Maternal and Child Health Care Hospital, Guangzhou, China; ^6^Department of Cancer Prevention Research, State Key Laboratory of Oncology in South China, Collaborative Innovation Center for Cancer Medicine, Sun Yat-sen University Cancer Center, Guangzhou, China

**Keywords:** developmental dyslexia, phonological loop, articulatory suppression, eye movement, reading

## Abstract

**Objective:** The study aimed to examine how the phonological loop influences reading ability and processing in Chinese children with developmental dyslexia (DD).

**Methods:** This study included 30 children with DD and 37 children without DD. Two types of articles (i.e., scenery prose and narrative story) and two conditions (under the conditions of articulatory-suppression and silent reading) were applied. An eye-link II High-Speed Eye Tracker was used to track a series of eye-movement parameters. The data were analyzed by the linear Mixed-Effects model.

**Results:** Compared with children without DD, Children with DD had lower reading achievement (RA), frequency of saccades (FS) and frequency of fixations (FF), longer reading time (RT) and average fixation duration (AFD), slower reading speed (RS), shorter average saccade amplitude (ASA) and fixation distance (FD), more number of fixations (NF), and number of saccades (NS). There were significant interactions between participant group and articulatory suppression on RT and FD. We also observed interaction effects between article types and articulatory suppression on RA, AFD, ASA, and FS.

**Conclusion:** Children DD exhibit abnormal phonological loop and eye movements while reading. The role of articulatory suppression on reading varies with the presentation of DD and the article type.

## Introduction

Developmental dyslexia (DD) is a neurodevelopmental disorder characterized by difficulties in accurate or fluent word recognition and spelling despite preserved intelligence, intact sensory abilities, and adequate instruction (1). Globally, 3.6~17.5% of students suffer from dyslexia (2); the prevalence of dyslexia among school-aged children was 4–10% in China (3–5).

As a slave system of working memory, the phonological loop is important to maintain and manipulate speech-based information. Multiple Western studies have indicated that children with DD have been found to exhibit impairments in the phonological loop (6–9), which was even posited that as a core deficit in DD (6). As a logographic language, Chinese uses square-shaped characters that each written form in Chinese is associated with a morpheme. A few studies showed that orthography rather than the phonological loop plays a dominant role in reading Chinese (10). However, a few research has indicated that Chinese children with DD exhibit abnormal phonological loop (9–12), suggesting that phonological loop impairment is universal across languages and orthographic systems.

The phonological loop recruits phonological stores and an articulatory rehearsal process (13). Phonological stores can hold memory traces for a few seconds, while the articulatory rehearsal process can prevent the decay of material stored in the phonological store by successively refreshing memory traces (14, 15). The phonological loop was typically measured by the tasks to recall a sequence of verbal items (e.g., digits, letters, and words) in the order in which they were presented. However, these tasks (e.g., the Digit Span task) only reflect the phonological stores rather than an articulatory rehearsal process, which could be assessed under articulatory suppression. Articulatory suppression refers to the disruptive effect of the articulation of irrelevant information during a verbal task on the normal functioning of the phonological loop (14, 15). Specifically, the articulation nongermane information prevents the articulatory control process from being used for rehearsal and visually presented items from being translated into a form that can enter the phonological store (16–18).

The literature has shown that articulatory suppression exerts differential effects on reading (19, 20). While its influence on simple-sentence comprehension is minimal, it can significantly affect the understanding of complex sentences (21–23). In addition, previous research has demonstrated that the effect of articulatory suppression on the accuracy and fluency of reading varied predictably not only by the difficulty of the text but also by the reading proficiency of readers. For instance, Millar found that articulatory suppression had no effect on either the accuracy or fluency of reading of easy texts among the most proficient readers, while among novice readers, the accuracy and fluency of reading decreased significantly (20). It has been demonstrated that children with DD exhibit deficits in reading proficiency. Therefore, we speculate that articulatory suppression may affect the reading ability of children with DD and the influence might vary when reading different types of articles.

Up to now, eye-movement technology is the most useful method to study reading ability. compared to traditional behavioral parameters, such as frequency or accuracy, eye-movement parameters can provide more precise information related to deeper cognitive activities during reading on the basis of ensuring the ecological validity of the research. The parameters of eye movements, such as fixation durations and saccadic amplitude, can provide important information on moment-to-moment processing (24). Fixation plays a central role in vision during both the acquisition and processing of visual information (25), and saccades allow for a new region of text to be brought into foveal vision for detailed analysis (24). Numerous studies showed that children with DD exhibit longer duration of fixations, shorter saccades and thus more fixations in reading than non-DD children (26–30). Our previous research showed that Chinese DD children had shorter saccade amplitude and mean saccade distance during non-reading tasks such as picture perception, visual search, Stroop and rapid-automatized-naming (RAN) tests (31–34). Unfortunately, the eye-movement characteristics of reading under articulatory suppression among Chinese children with DD remain unknown.

Based on the above speculation, we applied an eye-movement experiment to determine how articulatory suppression influences reading ability in Chinese children with DD. Addressing this is important to expand knowledge concerning the role of the phonological loop in the pathogenesis of DD in Chinese children, and provide evidence to establish efficient interventions for Chinese children with DD.

## Materials and Methods

### Subjects

This study included 30 children with DD (experimental group; 22 boys and 8 girls; mean age, 9.5 ± 1.2 years), and 37 without (control group; 24 boys and 13 girls; mean age, 9.1 ± 1.0 years). There were no significant differences between the case and control groups on age, gender, and grade.

We, at the beginning, recruited 673 students from the second to the fifth grade (mean age, 9.75 ± 1.17 years) in a primary school in Guangzhou, China. Written informed consent and assent has been obtained from all the subjects' parents. A series of procedures were applied to screen children with DD and their matched controls. We used the Pupil Rating Scale Revised-Screening for Learning Disabilities (PRS) to screen Children with DD (35). Non-verbal IQ was assessed by Raven's Standard Progressive Matrices (36). Reading achievement was assessed with the Character Recognition Test Battery and Assessment Scale for Primary School Children (CRTB), which is a widely-used, standardized written vocabulary test in mainland China that features both high reliability (0.98) and validity (0.98) (37).

Children with DD met the following inclusion criteria: (1) PRS score of < 65; (2) non-verbal IQ of ≥ 80; and (3) The CRTB score should be no more than the 2 standard deviations of the average grade level [≤ (mean−2SD)]. In the 673 students, there were 31 children met the inclusion criteria of DD, however, one subject with DD did not complete the eye-movement task, so this subject was not included in the analysis.

Non-DD children met the following inclusion criteria: (1) PRS scores of ≥ 65; (2) non-verbal IQ of ≥ 80; and (3) The CRTB score should be greater than the 2 standard deviations of the average grade level (>[mean−2SD]).

According to the reports provided by the parents and head-teachers of the study participants, none of the children both in the experimental and control groups had educational deprivation, suspected brain damage, uncorrected sensory impairment, or serious emotional or behavioral problems. All subjects had normal visual acuity and no subjects wore glasses.

#### The Pupil Rating Scale Revised-Screening for Learning Disabilities

It is a scale compiled by Myclebus and revised by Jingjin for screening children with learning disabilities (35). The scale has 24 items and is divided into five functional areas of verbal and non-verbal types: auditory comprehension and memory, language, time and orientation judgment, movement, and social behavior. The questionnaire has good reliability (retest correlation coefficients over 0.80) and fair validity (criterion validity correlation coefficients from 0.53 to 0.63).

#### Raven's Standard Progressive Matrices

Chinese researchers combined the first three units (A, A_B_, and B) of Raven' Color Progressive Matrices and the last three units (C, D, and E) of Raven' Advanced Progressive Matrices, forming the Combined Raven's Test (36). The Combined Raven's Test (CRT) has a retest stability of 0.95 and a validity of 0.56.

#### The Character Recognition Test Battery and Assessment Scale for Primary School Children

CRBT compiled by Wang and Tao of East China Normal University (37). In the test paper, there were 10 sets of questions, and each group had 6 to 33 Chinese characters. Students wrote combined words with presented Chinese characters in a limited time. The difficulty varies in different grades. According to the accuracy rate of combined words, the literacy ability was computed.

### Eye-Movement Recording

Eye movements were recorded with an Eyelink II High Speed Eye Tracker (SR Research Ltd., Canada), which features a high-resolution tracker (noise-limited at <0.01° root mean square; spatial resolution, <0.005°; 500 Hz binocular eye tracking) and few gaze-range errors of <0.5°. The participants were seated on a modified office chair that prevented rotational movement and were ~70 cm from a computer screen. Stimuli were displayed on a 53 mm (21-inch) liquid crystal display monitor with an average photo illumination of 200.00 lx. A brief nine-point calibration was performed prior to the experiment and repeated, if necessary, between blocks. Each trial was preceded by a short drift-correction procedure.

### Reading Task

Five short articles were used in the present study. All articles were selected from a second-grade reading book: “80 Chinese reading exercises in primary schools” (38). Article 0 (A0) was a narrative story used for habituation. The other four articles (A1~4) were used as test sets. Articles 1 and 3 were samples of scenery prose, while articles 2 and 4 were narrative stories. Article 1 had 127 words, 3 paragraphs, and 10 lines; article 2, 127 words, 2 paragraphs, and 8 lines; article 3, 138 words, 3 paragraphs, and 8 lines; and article 4, 159 words, 3 paragraphs, and 9 lines. All articles featured titles. Each article was followed by four sentences sourced from the article that the participants were asked to judge as “right or wrong”: an original sentence from the text, one with a different meaning from that of the original sentence, one in which a word was replaced by a homophone (景物[scenery] vs. 井屋[well house]), and one in which a word was replaced by characters with similar forms (碎[broken] vs. 醉[drunk]). These four types of sentences were presented in a random order after each article. All materials were presented in black on a white background. The font size of all Chinese characters was the same (1 cm × 1 cm), and the visual angle of each Chinese character was presented at 0.8°. According to the oral report, all participants had not previously been exposed to these reading exercises.

### Design and Procedures

In our study, the factors studied included group (children with DD vs. children without DD), articulatory suppression (yes vs. no), and article type (scenery prose vs. narrative story). During formal experiments, each subject needed to read four articles. Articles 1 and 2 were read silently without articulatory suppression, while articles 3 and 4 were read with articulatory suppression. Under the condition of articulatory suppression, the participants were required to continuously say “1+1 = 2” aloud. In order to preclude the influence of multiple readings on outcomes, all articles could only be read one time from beginning to end as quickly as possible. The children were then required to answer the four judgment sentences. Before experiments, both in the silent and articulatory suppression conditions, all subjects needed to practice using article 0 to ensure that they understood the test requirements.

### Data Analysis

#### Reading Comprehension Parameters

1. Reading Achievement (RA): Each of the four articles was followed by four true-or-false questions (1 point per question). The total RA score per article was 4.
2. Reading Time (RT): Because the word count of each article was different, we used reading time/100 words as the metric of RT: reading time/100 words = (total reading time in an article/the word count in the article) × 100.
3. Reading Speed (RS): the number of words the participant read per second: RS = the number of words in the article/total time spent reading the article.

#### Eye Movement Parameters

We used the following parameters to measure the eye movement (33).

1. Average Fixation Duration (AFD): average duration (in milliseconds) of all fixations.
2. Average Saccade Amplitude (ASA): average size (in degrees of visual angle) of the saccades.
3. Number of Fixations (NF): total number of fixations.
4. Number of Saccades (NS): total number of saccades.
5. Frequency of Fixations (FF): number of fixations per millisecond.
6. Frequency of Saccades (FS): number of saccades per millisecond during the trial.
7. Fixation Distance (FD): average distance between contiguous fixations in a trial (in degrees of visual angle).

### Statistical Analysis

The eye tracker recorded all the eye parameters and the response time when participants were reading. The results of the eye-movement experiments were extracted using the Eye-movement Analysis Software of the Eye Tracker. SAS statistical software (version 9.3) was used to analyze data. Chi-square test was used for categorical variables and *t*-test was used for continuous variables to compare characteristics between children with DD and those without. The linear Mixed-Effects model was used to test the main effects of group (children with DD vs. children without DD), articulatory suppression (yes vs. no), and article types (scenery prose vs. narrative story), as well as the interactions among groups, articulatory suppression, and article types. *p*-values of <0.05 were considered statistically significant.

## Results

### Sample Characteristics

Participant characteristics were shown in Table 1. Children with DD had lower scores of PRS and CRTB than children without DD (*P* < 0.001), while they were comparable in age, gender, and grade (*P* > 0.05).

**Table 1:** Characteristics of children with and without DD (*N* = 67).

**Characteristics**	**DD (*****N*** **= 30)**		**Non-DD (*****N*** **= 37)**		***t/X* ^**2**^**	***p***
	***n* (%)**	**Mean (SD)**	***n* (%)**	**Mean (SD)**		
Age		9.5 (1.2)		9.1 (1.0)	1.66	0.100
Gender (male)	22 (73.3)		24 (64.9)		−0.74	0.461
Grade						
2nd	6 (20.0)		11 (29.7)		−0.65	0.514
3rd	16 (53.4)		17 (46.0)			
4th	4 (13.3)		4 (10.8)			
5th	4 (13.3)		5 (13.5)			
PRS		56.5 (17.6)		98.6 (18.6)	**−7.90**	**<0.001**
CRTB		1,418.7 (517.1)		2,093.0 (638.0)	**−4.45**	**<0.001**

*DD, developmental dyslexia; PRS, The pupil rating scale revised screening for learning disabilities; CRTB, The Character Recognition Test Battery and Assessment Scale for Primary School Children. Chi-square test for categorical variables and t-test for continuous variables. Significant results are marked in bold*.

### Reading Comprehension Parameters

DD had significant main effects on RA (*F* = 24.71, *P* < 0.001), RT (*F* = 23.88, *P* < 0.001), and RS (*F* = 10.67, *P* = 0.001). Compared to children without DD, those with DD had a lower RA, longer RT, and slower RS.

Articulatory suppression exerted main effects of only marginal significance on RA (*F* = 3.18, *P* = 0.076), RT (*F* = 2.97, *P* = 0.086), and RS (*F* = 3.28, *P* = 0.071). Compared to silent reading, the articulatory suppression condition was associated with higher RA, shorter RT, and faster RS.

Article type also had significant main effects on RA (*F* = 11.93, *P* < 0.001), RT (*F* = 7.65, *P* = 0.006), and RS (*F* = 8.16, *P* = 0.005). Compared to narrative story reading, all children had lower RA, longer RT, and slower RS in scenery prose reading.

**Figure 1:**
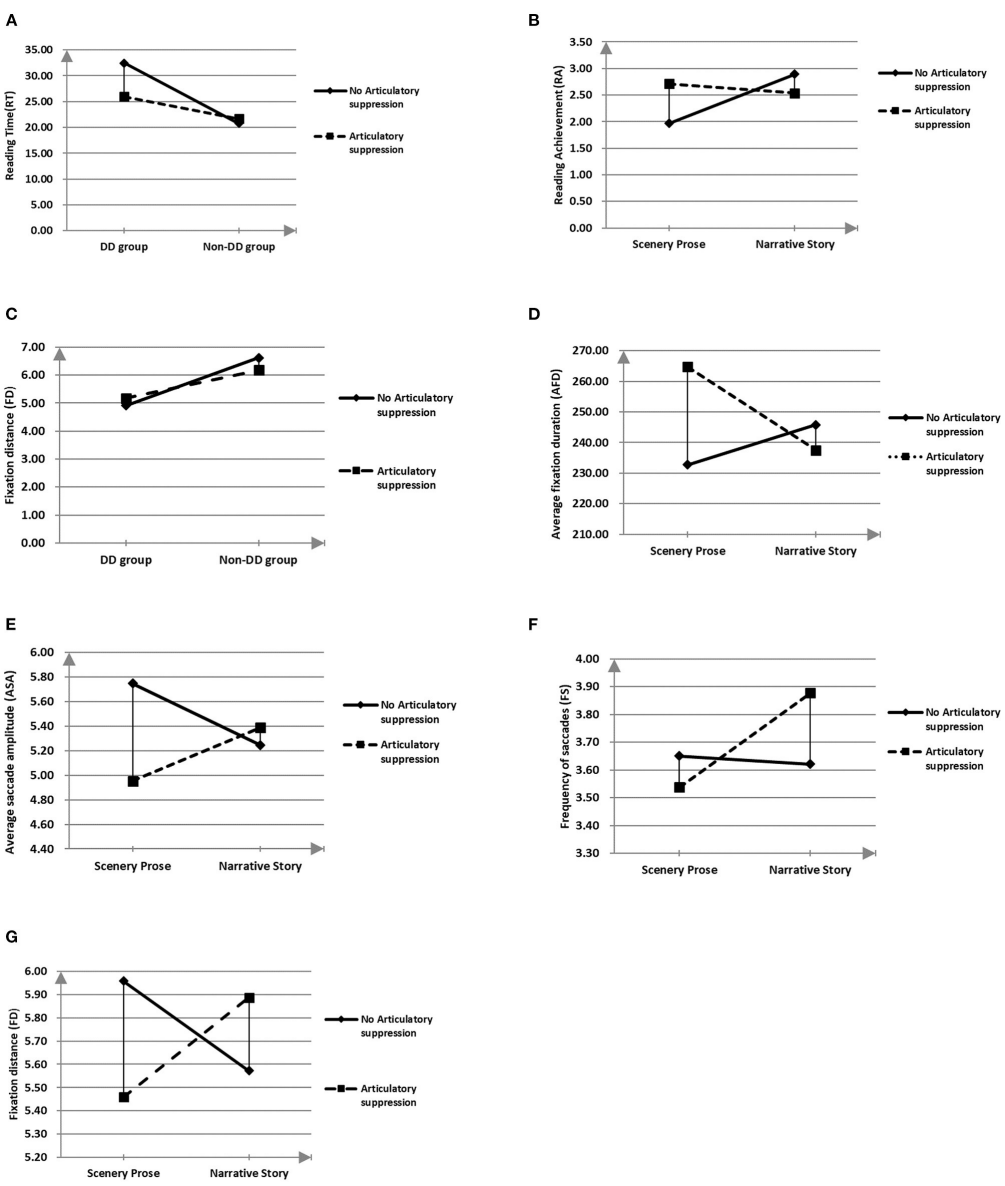
Significant interactions among group, articulatory suppression, and article type on eye-movement and reading parameters. **(A)** Demonstrates a significant interaction between DD and articulatory suppression on RT. **(B)** Demonstrates a significant interaction effect between article type and articulatory suppression on RA. **(C)** Demonstrates a significant interaction between groups and articulatory suppression on FD. Significant interactions were also found between articulatory suppression and article types on AFD **(D)**, ASA **(E)**, FS **(F)**, and FD **(G)**. DD, Developmental dyslexia; RA, Reading achievement; FD, Fixation distance; AFD, Average fixation duration; ASA, Average saccade amplitude; FS, Frequency of saccades.

Interactions among groups, articulatory suppression, and article type on reading comprehension parameters are shown in Table 2. A significant interaction was found between DD and articulatory suppression on RT (P for interaction, 0.025; Figure 1A). Relative to silent reading, articulatory suppression was associated with significant decreases in the RT of Children with DD and no significant change among non-DD children. We also found a significant interaction effect between article type and articulatory suppression on RA (Figure 1B). RA decreased during scenery prose reading with articulatory suppression and RA increased during narrative story reading.

**Table 2:** Interactions among group, articulatory suppression, and article type on parameters of reading comprehension.

**Reading comprehension parameters**	**Mean (SD)**						**Interactions**							
	**Groups (1)**		**Articulatory suppression (2)**		**Article types (3)**		**1 × 2**		**1 × 3**		**2 × 3**		**1 × 2 × 3**	
	**DD**	**Non-DD**	**No**	**Yes**	**Scenery** **prose**	**Narrative** **story**	***F***	***P***	***F***	***P***	***F***	***P***	***F***	***P***
RA	2.26 (0.08)	2.80 (0.07)	2.43 (0.08)	2.62 (0.08)	2.34 (0.08)	2.72 (0.08)	0.50	0.479	0.28	0.594	**25.96**	**<0.001**	0.41	0.524
RT	29.22 (1.21)	21.24 (1.09)	26.63 (1.15)	23.82 (1.15)	27.48 (1.15)	22.97 (1.15)	**5.10**	**0.025**	0.38	0.538	0.68	0.412	0.02	0.878
RS	4.74 (0.27)	5.91 (0.24)	5.00 (0.25)	5.64 (0.25)	4.81 (0.25)	5.83 (0.25)	0.42	0.518	0.11	0.737	1.20	0.275	0.64	0.426

*DD, development dyslexia; RA, Reading Achievement; RT, Reading Time; RS, Reading Speed. Significant results are marked in bold*.

### Eye-Movement Parameters

Interactions among groups, articulatory suppression, and article type on eye-movement parameters are shown in Table 3. DD had significant main effects on AFD (*F* = 11.84, *P* < 0.001), ASA (*F* = 60.71, *P* < 0.001), NF (*F* = 6.14, *P* = 0.014), NS (*F* = 19.62, *P* < 0.001), FS (*F* = 10.42, *P* = 0.001), FF (*F* = 35.43, *P* < 0.001), and FD (*F* = 57.38, *P* < 0.001). Compared to the control group, Chinese children with DD had a significantly longer AFD, shorter ASA and FD, higher NF and NS, and lower FS and FF.

**Table 3:** Interactions among group, articulatory suppression, and article type on eye-movement parameters.

**Eye-movement parameters**	**Mean (SD)**						**Interactions**							
	**Groups (1)**		**Articulatory suppression (2)**		**Article types (3)**		**1 × 2**		**1 × 3**		**2 × 3**		1*2*3	
	**DD**	**Non-DD**	**No**	**Yes**	**Scenery** **prose**	**Narrative** **story**	***F***	***P***	***F***	***P***	***F***	***P***	***F***	***P***
AFD	256.34 (4.82)	234.01 (4.34)	239.27 (4.59)	251.08 (4.59)	248.72 (4.59)	241.63 (4.59)	1.96	0.163	1.94	0.165	**9.63**	**0.002**	0.00	0.992
ASA	4.64 (0.13)	6.03 (0.12)	5.50 (0.13)	5.17 (0.13)	5.35 (0.13)	5.32 (0.13)	2.58	0.109	0.22	0.639	**6.83**	**0.010**	0.00	0.972
NF	111.66 (4.52)	96.59 (4.07)	105.11 (4.30)	103.14 (4.30)	108.28 (4.30)	99.98 (4.30)	1.75	0.187	0.36	0.549	<0.01	0.987	1.43	0.232
NS	132.68 (4.61)	105.23 (4.15)	117.59 (4.38)	120.32 (4.38)	124.85 (4.38)	113.06 (4.38)	1.65	0.200	0.11	0.741	1.15	0.284	0.34	0.563
FS	3.53 (0.07)	3.82 (0.06)	3.64 (0.06)	3.71 (0.06)	3.59 (0.06)	3.75 (0.06)	1.22	0.270	0.65	0.421	**4.28**	**0.040**	0.53	0.469
FF	2.95 (0.06)	0.063 (0.06)	3.28 (0.06)	3.12 (0.06)	3.14 (0.06)	3.26 (0.06)	0.74	0.390	1.63	0.203	0.38	0.536	3.64	0.058
FD	5.04 (0.13)	6.40 (0.12)	5.77 (0.13)	5.67 (0.13)	5.71 (0.13)	5.73 (0.13)	3.97	**0.047**	0.26	0.611	**5.14**	**0.024**	0.15	0.701

*DD, developmental dyslexia; AFD, Average fixation duration; ASA, Average saccade amplitude; NF, Number of fixations; NS, Number of saccades; FS, Frequency of saccades; FF, Frequency of fixations; FD, Fixation distance. Significant results are marked in bold*.

Articulatory suppression had only marginally significant main effects on AFD (*F* = 3.31, *P* =0.070), ASA (*F* = 3.35, *P* = 0.068), and FF (*F* = 3.19, *P* = 0.075), but not on NF (*F* = 0.10, *P* = 0.747), NS (*F* = 0.19, *P* = 0.659), FS (*F* = 0.64, *P* =0.423) or FD (*F* = 0.26, *P* = 0.609). Articulatory suppression lengthened AFD, shortened ASA, and diminished FF.

Article type had a marginally significant main effect on NS (*F* = 3.62, *P* = 0.058) and FS (*F* = 3.01, *P* = 0.084), but not on AFD (*F* = 1.19, *P* = 0.276), ASA (*F* = 0.03, *P* = 0.853), NF (*F* = 1.86, *P* = 0.174), FF (*F* = 2.26, *P* = 0.134), or FD (*F* = 0.01, *P* = 0.907). Compared to narrative story reading, scenery prose reading tended to be associated with higher NS and lower FS.

There was a significant interaction between groups and articulatory suppression on FD (Figure 1C). Compared to silent reading, articulatory suppression was associated with an increase in FD in the Children with DD and a decrease in FD in the non-DD group. Significant interactions were also found between articulatory suppression and article types on AFD (Figure 1D), ASA (Figure 1E), and FS (Figure 1F), FD (Figure 1G). Across all participants, AFD significantly increased, and ASA, FS and FD significantly decreased during scenery prose reading with articulatory suppression relative to silent scenery prose reading. The reading of narrative stories, however, was associated with a reduced AFD, and increased ASA, FS and FD with articulatory suppression relative to silent narrative story reading; these differences were non-significant.

## Discussions

Our findings expand the knowledge about the distinctive performance of phonological loop in children with DD, which might be a target for establishing effective interventions in the future.

First, compared to silent reading, imposing articulatory suppression during reading significantly decreased the RT and increased the FD of children with DD, and tended to increase the RT and decrease the FD of children without DD. FD refers to the distance between contiguous fixations, an important parameter of visual attention span defined by the number of characters that a reader processes during a fixation. Reduced FD means that it is more difficult to process the reading material (24, 39, 40). The role of articulatory suppression on reading is relatively complex. It may effectively inhibit the effect of speech coding, decrease reading scores, lengthen reading time, and lower accuracy (41). However, not all individuals may naturally adopt a strategy of phonological recoding or rehearsal while reading (42). Baddeley posited that neuropsychological patients with impaired phonological store make little-to-no use of their defective phonological short-term store (23), and he thought that participants may abandon the loop when recall becomes difficult, e.g., when a list lengthens (43). In the current study, children with DD exhibit lower RA. Accordingly, we speculate that as children with DD exhibit impairments of the phonological loop, they may adopt a strategy divergent from that of phonological recoding or rehearsal under articulatory suppression, abandonment of the phonological loop during articulatory suppression reduces RT and increases FD. By contrast, children without DD ensure precise comprehension of articles by increasing RT and decreasing FD under articulatory suppression.

Second, our results indicated that the effect of articulatory suppression varies according to article type: it had little influence on simple narrative story comprehension but interfered significantly with simple scenery prose comprehension. The literature has shown that articulatory suppression can significantly disrupt the normal functioning of the phonological loop. However, this effect varies predictably with the difficulty of text, because participants, like children with DD, may abandon the phonological loop when phonological code and recall becomes difficult (17, 21, 44). We found that all children exhibited higher RA, shorter RT, and faster RS, less NS, and higher FS during narrative story reading than scenery prose reading, suggesting that the narrative stories were easier to understand than the scenery prose. We concluded that articulatory suppression has little influence on the reading of simple narrative stories. However, this may be attributable to the simple narrative story selected in our research having featured clear logic, accessible clues, and applicability to everyday common sense that rendered the involvement of the phonological loop unnecessary. On the contrary, the scenery prose features rich content and an uneven distribution of information, rendering the application of common sense to better understand and recall the text's meaning difficult. Moreover, scenery prose requires rehearsal and recruitment of the phonological store to retain, understand, and extract a large amount of information contained therein. Combined with previous studies, the effect of articulatory suppression on simple-sentence comprehension is minimal, while its effect on the understanding of complex sentences is significant (21–23), we believe that the effect of articulatory suppression varies according to the complexity of reading materials but not article type. The more complex the reading materials are, the more memory store is needed, and the greater the effect of articulatory suppression on reading processing is.

### Strength and Limitations

It was the first time to use eye-movement technological to study the effect of articulatory suppression on the reading processing of children with DD. We acknowledge that the current study has several limitations. Firstly, as a case-control study, this investigation could not provide proof of causal relationships among abnormal eye movements, impairments of the phonological loop, and dyslexia. Secondly, in diagnosis, we assess reading level by vocabulary test. However, vocabulary test is the determinant of reading level for primary school students, and is most commonly used to assess the reading level of Chinese children (45, 46). Thirdly, the reading tasks used in the current study were chosen from a second-grade reading book, which might be a little easy for some children in higher grades. However, most participants in this study were in grade 2 and 3. Therefore, it should be appropriate to have a second-grade reading materials to make sure they all can read, especially for children with DD. Future studies applying varied reading difficulties are needed. Finally, the article types just included scenery prose and narrative story, so the results couldn't reflect the processing characteristics of the other article types in children with DD.

## Conclusions

In the current study, we found that Chinese children with DD exhibit impairments in abnormal eye movements when reading whether or not under articulatory suppression. Under articulatory suppression, Chinese children with DD showed increased FD and reading speed, while children without DD had decreased FD and reading speed. Articulatory suppression has little influence on the reading of simple narratives but significantly interferes with that of scenery prose. More studies are needed to confirm our findings and further explore the effects of varied difficulties and types of reading materials on reading.

## Data Availability Statement

The original contributions presented in the study are included in the article, further inquiries can be directed to the corresponding author.

## Ethics Statement

The studies involving human participants were reviewed and approved by the Medical Ethics Committee of Sun Yat-sen University. Written informed consent to participate in this study was provided by the participants' legal guardian/next of kin.

## Author Contributions

WL, BL, JZ, JM, CX, and JW: methodology, formal analysis, and writing-reviewing and editing. JJ: conceptualization, validation, writing-reviewing and editing, and supervision. XL: conceptualization, methodology, investigation, formal analysis, writing-original draft, validation, writing-reviewing and editing, supervision, project administration, and funding acquisition. All authors contributed to the article and approved the submitted version.

## Funding

This work was supported by Key-Area Research and Development Program of Guangdong Province (grant number 2019B030335001) and National Natural Science Foundation of China (grant number 81673197). The funding sources had no role in the design of the study, data collection, analysis, interpretation of data nor the writing of the manuscript.

## Conflict of Interest

The authors declare that the research was conducted in the absence of any commercial or financial relationships that could be construed as a potential conflict of interest.

## Publisher's Note

All claims expressed in this article are solely those of the authors and do not necessarily represent those of their affiliated organizations, or those of the publisher, the editors and the reviewers. Any product that may be evaluated in this article, or claim that may be made by its manufacturer, is not guaranteed or endorsed by the publisher.
